# Flexible and efficient perovskite quantum dot solar cells via hybrid interfacial architecture

**DOI:** 10.1038/s41467-020-20749-1

**Published:** 2021-01-20

**Authors:** Long Hu, Qian Zhao, Shujuan Huang, Jianghui Zheng, Xinwei Guan, Robert Patterson, Jiyun Kim, Lei Shi, Chun-Ho Lin, Qi Lei, Dewei Chu, Wan Tao, Soshan Cheong, Richard D. Tilley, Anita W. Y. Ho-Baillie, Joseph M. Luther, Jianyu Yuan, Tom Wu

**Affiliations:** 1 School of Materials Science and Engineering, University of New South Wales (UNSW), Sydney, NSW 2052 Australia; 2 Institute of Functional Nano & Soft Materials (FUNSOM), Jiangsu Key Laboratory for Carbon-Based Functional Materials & Devices, Soochow University, 215123 Suzhou, Jiangsu People’s Republic of China; 3 School of Engineering, Macquarie University, Sydney, NSW 2109 Australia; 4 School of Materials Science and Engineering, Nankai University, Tianjin, 300350 People’s Republic of China; 5 National Renewable Energy Laboratory, Golden, CO 80401 USA; 6 Australian Centre for Advanced Photovoltaics, University of New South Wales, Sydney, NSW Australia; 7 School of Physics, University of Sydney Nano Institute, The University of Sydney, Sydney, NSW 2006 Australia; 8 Electron Microscope Unit, Mark Wainwright Analytical Centre, UNSW, Sydney, NSW 2052 Australia

**Keywords:** Solar cells, Solar cells, Quantum dots

## Abstract

All-inorganic CsPbI_3_ perovskite quantum dots have received substantial research interest for photovoltaic applications because of higher efficiency compared to solar cells using other quantum dots materials and the various exciting properties that perovskites have to offer. These quantum dot devices also exhibit good mechanical stability amongst various thin-film photovoltaic technologies. We demonstrate higher mechanical endurance of quantum dot films compared to bulk thin film and highlight the importance of further research on high-performance and flexible optoelectronic devices using nanoscale grains as an advantage. Specifically, we develop a hybrid interfacial architecture consisting of CsPbI_3_ quantum dot/PCBM heterojunction, enabling an energy cascade for efficient charge transfer and mechanical adhesion. The champion CsPbI_3_ quantum dot solar cell has an efficiency of 15.1% (stabilized power output of 14.61%), which is among the highest report to date. Building on this strategy, we further demonstrate a highest efficiency of 12.3% in flexible quantum dot photovoltaics.

## Abstract (web summary)

Perovskite quantum dots film has better mechanical stability and structural integrity compared to bulk thin film. Here, the authors demonstrate higher endurance of quantum dot films and develop hybrid CsPbI3 QD/PCBM device with PCE of 15.1% and 12.3% on rigid and flexible substrates, respectively.

## Introduction

Colloidal quantum dots (QDs) have remarkably tunable properties such as size-dependent absorption and emission wavelengths and energy levels, exhibit efficient multiple exciton effects, and compared to other solution-processed materials show decent stability^1–8^. These unique features facilitate their wide applications in optoelectronic devices such as photodetectors^9–12^, light-emitting diodes^13,14^, and photovoltaics^15–17^. The best PbS QD solar cell has achieved a power conversion efficiency (PCE) of 13.8% due to improvements in surface passivation and device structure^18^. Concurrently, all-inorganic CsPbI_3_ QDs have emerged as a rising star for photovoltaic applications because surface strain enables QDs to retain the perovskite phase, whereas thin films are metastable and convert readily to the non-perovskite phase. QDs exhibit high photoluminescence (PL) quantum yields due to impressive defect tolerance^19–22^, which translates into high open-circuit voltages. Advances in CsPbI_3_ QD solar cells have enabled high efficiency over 15% to be achieved, showing great potential for photovoltaics^23,24^. Importantly, fabrication of perovskite QDs involves colloidal synthesis and processing using a variety of more common nonpolar organic solvents such as hexane, octane, or toluene at room temperature, whereas thin-film perovskites are normally processed from polar aprotic solvents such as *N*,*N*-dimethylformamide, which is quite toxic, opening a new platform for developing high-performance QD optoelectronic devices^25–29^.

In addition to this, QD materials, with intrinsic nanoscale dimensions, offer an additional and underappreciated characteristic of mechanical flexibility without performance loss^30,31^. Unfortunately, there is barely any experimental evidence proving their mechanical endurance relative to thin films, especially for efficient flexible photovoltaics. We believe that there are two major merits that flexible QD devices can offer. One is high-quality QD film can be deposited at room temperature, which is ideal for flexible substrates^32–35^. The other is QDs can deliver a high power output-per-weight, necessary for lightweight and portable devices, spacecraft, etc^36^. Although previous studies have demonstrated excellent flexural endurance of QD photovoltaics^37,38^, none have demonstrated high PCEs, probably due to poor charge transfer and carrier extraction efficiencies at QD heterointerfaces and interfaces. PCEs of flexible QD photovoltaics have been limited to below 10%^38^, which lag significantly behind flexible organic and thin-film perovskite solar cells^39,40^.

In this work, we have overcome these limitations demonstrating efficient flexible QD photovoltaics using emerging CsPbI_3_ perovskite QDs. Taking advantage of extremely high surface areas of QDs, we developed a thin hybrid interfacial architecture (HIA) by introducing phenyl-C61-butyric acid methyl ester (PCBM) into the CsPbI_3_ QD layer. The PCBM bonds with the under-coordinated Pb^2+^ ions on the QD surfaces through functional carboxyl groups and creates an exciton cascade between the CsPbI_3_ QD layer and the electron transport layer (ETL) SnO_2_, thus enabling efficient charge transfer and also promoting exciton dissociation at both QD heterointerfaces and QD/ETL interfaces. Using this HIA strategy, we achieve a champion PCE of 15.1% (stabilized power output of 14.61%). This HIA enables flexible QD solar cells with better adhesion to the ETL showing the highest efficiency of 12.3% with improved mechanical stability confirmed by detailed morphological characterization compared to thin films with much larger grains.

## Results

### PCBM/CsPbI_3_ QD hybrid film

**Fig. 1:**
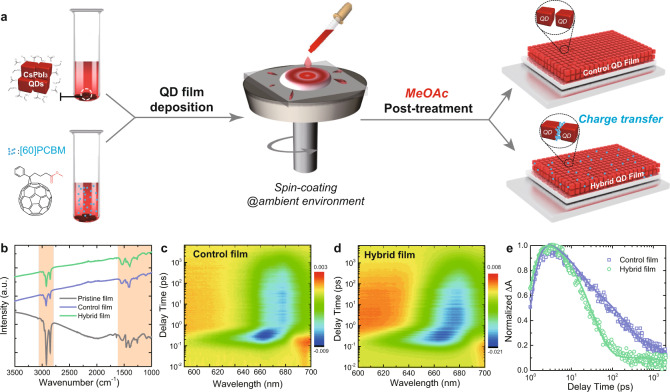
The preparation and properties of CsPbI_3_ QD and CsPbI_3_ QD/PCBM films. **a** Schematic of the fabrication of pristine, control and hybrid CsPbI_3_ QD films. **b** FTIR spectra of pristine, control, and hybrid films on SnO_2_/ITO glass substrates (characteristic peaks of organic species are highlighted by the red background). **c**, **d** 2D time-wavelength-dependent TA color maps of the control and the hybrid CsPbI_3_ QD films excited at 600 nm, the excitation fluence is 4.2 µJ/cm^2^/pulse. **e** Normalized TA dynamics of the ground-state bleach (GSB: 670 nm) for control and hybrid CsPbI_3_ QD films. For the control film, the components are *τ*_1_ = 1.3 ps, *τ*_2_ = 52.3 ps, and *τ*_3_ = 483.2 ps. For the hybrid film, the time components are extracted as *τ*_1_ = 1.4 ps, *τ*_2_ = 57.6 ps, and *τ*_3_ = 312.8 ps.

CsPbI_3_ QDs were synthesized and purified according to the procedures previously published^19^. PCBM was added into the CsPbI_3_ QDs dispersed in chlorobenzene (CB) to obtain a hybrid solution. The CsPbI_3_ QDs with and without PCBM have a similar cubic shape with an average size of about 10 nm, characterized by the transmission electron microscopy (TEM) shown in Supplementary Fig. 1. Figure 1a depicts two different types of QD film deposition and solid-state ligand removal process. In general, the QD solution was spin-coated onto a substrate under ambient conditions with low relative humidity (RH < 10%) at room temperature. Then the native long oleate ligands were removed by soaking the as-cast QD film in anhydrous methyl acetate (MeOAc)^20^. This procedure was repeated 3–5 times to build up ~300-nm-thick films.

We first characterized the properties of CsPbI_3_ QD films with or without PCBM using Fourier transform infrared (FTIR), femtosecond transient absorption (fs-TA), and PL spectroscopy. In the FTIR spectra (Fig. 1b), the signal intensities of C-H modes at 2851 and 2921 cm^−1^ for both control and hybrid films were greatly reduced compared to the pristine film (without MeOAc treatment), which indicates the degree to which the native long-chain ligands were removed after the MeOAc treatment and the addition of PCBM into the QD matrix had little effect on the process of surface ligand removal. To investigate the charge transfer dynamics in control and hybrid QD films, fs-TA measurements were carried out to collect detailed information for both radiative and nonradiative processes^41,42^. As shown in Fig. 1c, d, the hybrid film exhibits more obvious bleaching shifts at increasing delay time than the control, demonstrating improved charge separation in hybrid films. The corresponding time-resolved components extracted from the dynamics at 670 nm are shown in Fig. 1e. The time constants *τ*_1_, *τ*_2_, and *τ*_3_ calculated through a tri-exponential fitting are generally attributed to the hot phonon bottleneck, the Auger recombination, and the charge transfer process, respectively^43–45^. Clearly, the reduced charge transfer time in the hybrid film (312.8 ps) indicates more efficient charge extraction compared with that in the control film (483.2 ps). The slightly longer Auger recombination process in the hybrid film (57.6 ps vs. 52.3 ps) suggests lower trap density, which may be attributed to passivation by PCBM. In order to confirm this, we further performed space-charge-limited current (SCLC) measurements on the control and target films to investigate the density of surface defects. As shown in Supplementary Fig. 2, the trap density is decreased in the hybrid film relative to the control film, indicating PCBM passivation of the CsPbI_3_ QD surface defects.

Steady-state PL and time-resolved PL (TRPL) measurements were used to confirm the improved carrier dynamics observed in fs-TA measurements. We also investigate the PCBM/CsPbI_3_ QD planar film for a better comparison. As shown in Supplementary Fig. 3, compared to the pristine film, the control QD film exhibits a red-shifted PL peak, indicating improved electronic coupling after surface ligand removal^41^. In the PL decay profile (Supplementary Fig. 4), the PCBM/CsPbI_3_ QD hybrid film demonstrates the fastest decay, suggesting improvement of heterojunction structure (hybrid film) in electron extraction, indicating PCBM addition in the QD film could provide an effective transport channel for accelerating charge extraction, which is also consistent with the fs-TA analysis.

### Performance of CsPbI_3_ QD photovoltaics

**Fig. 2:**
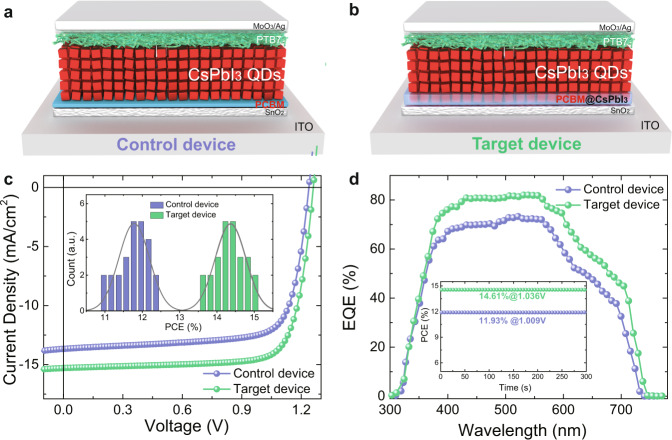
Photovoltaic performance of solar cells. **a**, **b** Schematic diagram of the control device and target device. **c** *J*–*V* curves measured under AM 1.5 G solar irradiation at 100 mW/cm^2^ (inset: PCE distribution of control and target devices). **d** EQE curves of control and target devices (inset: stabilized power output of control and target devices).

The schematic diagrams of the control and the target PCBM/CsPbI_3_ QD solar cell architectures are given in Fig. 2a, b, respectively. To fabricate the control devices without hybrid films, an ultra-thin PCBM was prepared on top of the SnO_2_ layer. Each layer of both devices is clearly identified in the cross-sectional SEM image presented in Supplementary Fig. 5. To achieve the best-optimized PV performance, as shown in Supplementary Table 1, we first tuned the thickness of the QD absorber layer for control QD devices. A decent efficiency of 12.4% is obtained using four depositing cycles. Then we optimized the thickness of the hybrid QD layer in the HIA, concluding the device with the 1:4 thickness ratios of hybrid QD layers to control QD layers delivered the highest efficiency up to 15.1%, which is among the highest reported efficiency for CsPbI_3_ QD solar cells (Supplementary Table 2). As shown in Fig. 2c and Table 1, the enhancement of PCE results from the significantly improved short-circuits current density (*J*_sc_). External quantum efficiency (EQE) was then carried out on the optimal control and target devices, showing the EQE of the target QD device is enhanced across the entire response region (Fig. 2d), which could result from the improved charge collection efficiency through introducing HIA rather than absorption improvement. The integrated *J*_sc_ from the EQE measurements is 12.7 and 15.1 mA/cm^2^ for the control and target device (Supplementary Fig. 6), respectively, which matches well with the corresponding *J*_sc_ obtained from the *J*–*V* curves. Furthermore, we increased the thickness of hybrid QD layers with the same control QD layers. The device having thicker hybrid QD layers exhibits decreased efficiency of 13.8% (Supplementary Table 1) that could have resulted from the unfavorable effects of CB solvent during upper hybrid layer deposition^46^, leading to PCBM exfoliation from CsPbI_3_ QDs in the bottom layer.

**Table 1:** Statistics of CsPbI_3_ QD solar cell performance with varied QD absorber layers based on 24 devices.

Substrate	Device structure	*V*_oc_ (V)	*J*_sc_ (mA/cm^2^)	FF	PCE (%)
Glass/ITO (rigid)	Control	1.23 (1.22 ± 0.02)	13.6 (13.3 ± 0.7)	0.74 (0.73 ± 0.02)	12.4 (11.9 ± 0.4)
	Target	1.26 (1.24 ± 0.03)	15.2 (14.4 ± 0.9)	0.78 (0.76 ± 0.03)	15.1 (14.6 ± 0.6)
PET/ITO (flexible)	Control	1.16 (1.11 ± 0.06)	12.0 (11.5 ± 1.6)	0.70 (0.67 ± 0.06)	9.7 (8.5 ± 0.6)
	Target	1.24 (1.19 ± 0.04)	13.6 (13.1 ± 1.2)	0.73 (0.72 ± 0.05)	12.3 (11.3 ± 0.4)

It should be noted that the HIA modification also leads to reduced *J–V* hysteresis (Supplementary Fig. 7). The stabilized power output of 14.61% for the target device and 11.93% for the control device were recorded (Fig. 2d), confirming the reliability of *J*–*V* measurements. We also characterized device stability (Supplementary Fig. 8) and found that the control device lost 50% of its initial efficiency while the target device degraded by only 30% after storage in the dry air-filled box for 14 days, indicating that the HIA is also beneficial for improving device stability. The contact angle of water drop on control and the hybrid film is presented in Supplementary Fig. 9, indicating the hybrid film has a more hydrophobic nature, which is beneficial for depositing the upper QD layer and improving the interfacial contact.

**Fig. 3:**
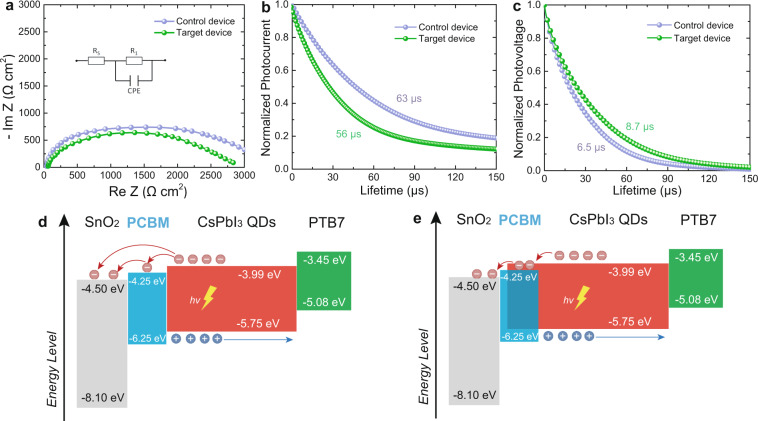
Characterization of QD solar cells. **a** EIS curves of champion control and target devices (inset: equivalent circuit). **b**, **c** TPV and TPC curves in champion control and target devices. **d**, **e** Band energy level diagram of the control and target devices.

To understand the enhancements of QD photovoltaic performances, additional device characterization was performed. Electrochemical impedance spectroscopy (EIS) was used to investigate the quality of interfaces. Analysis of the low-frequency region, including recombination chemical capacitance (*C*_rec_) and the recombination resistance (*R*_rec_), provides insight into the recombination processes, whereas analysis of the high-frequency region, including transport chemical capacitance (*C*_trans_) and the transport resistance (*R*_trans_), gives information about the charge transport processes. As shown in Fig. 3a, the target device with HIA presents smaller *R*_trans_ and larger *R*_rec_ than the control, indicating that the recombination process is effectively suppressed, which is consistent with the *J*–*V* measurements on the devices. Additionally, their different dark *J*–*V* characteristics in Supplementary Fig. 10 show that the target device exhibits a lower leakage current under reverse bias compared to the control device, indicating decreased carrier recombination. As shown in Supplementary Fig. 11, from the *J*_sc_ as a function of light intensity, we found there are few bimolecular recombinations in both systems, however, which is not dominant. It should be noted that the ideality factor of unity would also not always be due to bimolecular recombination, especially if there are space-charge regions in their device^47^. Using the HIA strategy can slightly decrease the bimolecular recombination. For *V*_sc_ as a function of light intensity, the values of the slope were calculated to be 1.29 and 1.43 kT/q for the target and control devices, respectively. The lower slope for the target based device suggests a reduction in trap-assisted carrier recombination, which is the dominant recombination in both systems. However, both values are slightly far from the ideal values (kT/q), and interpreting these measurements is a general method to investigate the recombination loss. Furthermore, transient photocurrent and photovoltage (Fig. 3b, c) were performed on both devices. The target solar cell had a shorter carrier lifetime of 56 μs while the control solar cell had a long carrier lifetime of 63 μs, suggesting that the carriers in the target device were transported more quickly due to PCBM introduction. The transient photovoltage exhibits a similar trend and the target device has a longer lifetime of 8.7 µs (6.5 µs for the control), demonstrating a faster charge transport rate. All these results indicate the intrinsic mechanism of enhancement of QD photovoltaic performance lies in the improvement of the QD/ETL interfaces, which is attributed to the improved energy level alignment. The values of the valence band (VB) energy level was characterized by ultraviolet photoelectron spectroscopy (UPS) (Supplementary Fig. 12), and their conduction bands (CB) were further calculated according to their optical bandgaps (Supplementary Fig. 13). As shown in Fig. 3d, e, PCBM has the appropriate electron affinity serving as a modification layer between the SnO_2_ and the CsPbI_3_ QDs, which leads to the suppressed charge recombination at the interface between SnO_2_ and the CsPbI_3_ QD layer. The efficient charge transfer in the hybrid device is enabled by the formation of a cascade CB alignment that efficiently injects the photogenerated electrons into the CB of PCBM and then to that of the SnO_2_ ETL, as evidenced by the fs-TA and PL measurements. Hence, PCBM could be seen as a fast electron transport channel in the target QD device, which leads to improved device performance.

### CsPbI_3_ QD-PCBM molecular interaction

**Fig. 4:**
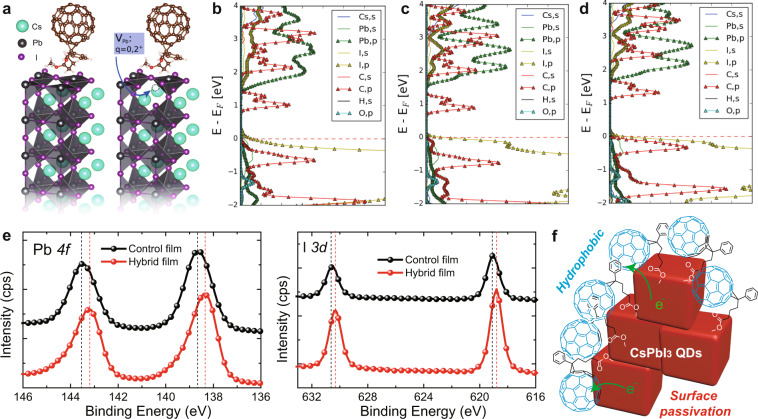
Molecular interaction between QD and PCBM. **a** Schematic diagrams of the simulated unit cell, showing the perovskite slab system with and without a charged Pb-vacancy defect. **b**–**d** The electronic pDOS for three cases: without Pb-vacancy, with a neutral, and with a Pb-vacancy carrying a formal 2+ charge. **e** XPS signals of Pb 4*f* and I 3*d* in the control and the hybrid films. **f** Schematic diagram of the hydrophobic PCBM passivating surface of CsPbI_3_ QDs.

To further understand the molecular interaction between CsPbI_3_ QD and PCBM, density functional theory (DFT)-based molecular dynamic simulation and X-ray photoelectron spectrum (XPS) measurements were carried out. First, surface defects in the perovskite may lead to trap states that not only impede charge transport between the light absorber and the PCBM but also increase unfavorable interfacial recombination^48,49^. As shown in Fig. 4a–d, Pb-vacancy (V_Pb_) on (100) surfaces is one potential defect type that has been shown in the bulk, leading to shallow levels near the valence band maximum (VBM), resulting in undesirable charge accumulation and potential recombination at the perovskite-PCBM interface. However, the projected density of states (pDOS) obtained from DFT simulations (PAW PBE-GGA) on a 2 × 2 × 4 perovskite slab with a PCBM molecule on the most commonly exposed (100) facet do not show detrimental electronic states for either charged or neutral V_Pb_ defects. The carbon *p*-states (C, *p*) of the PCBM are well aligned to receive electrons from the perovskites. The iodine *p*-levels that define the perovskite’s VBM do not encroach on the carbon *p*-levels for the relatively large concentration of V_Pb_ simulated (25% atomic V_Pb_/Pb), implying the perovskite is free of hole traps at the surface for this defect type. Second, it was found that the peaks of Pb 4*f* (Fig. 4e), I 3*d* (Fig. 4e), and Cs 3*d*/5 (Supplementary Fig. 14) shifted towards a lower binding energy level in CsPbI_3_ QDs of the hybrid film, suggesting the formation of coordination bonds between carboxyl moieties and mostly under-coordinated Pb^2+^ ions on QD surfaces. The systematic characterizations of the hybrid QD blend film indicate the improved carrier dynamic and molecular interaction. As illustrated in Fig. 4f, when PCBM blends with CsPbI_3_ QDs at the optimal ratio, the spherically symmetric C_60_ facilitates the efficient charge transfer at the interfaces. In addition, one problem with the conventional CsPbI_3_ QD film after the process of ligand removal is its sensitivity to moisture. The addition of hydrophobic PCBM could not only reduce the QD boundaries but also passivate the QD surfaces via forming strong bonding of the carboxyl group with the under-coordinated Pb^2+^ ions on the QD surfaces. To further confirm the bonding of the carboxyl group with the under-coordinated Pb^2+^ ions, we further performed FTIR characterization. As shown in Supplementary Fig. 15, the FTIR spectrum of PCBM possesses a strong peak located at ~1738 cm^−1^, which is attributed to the stretching vibration of the C=O group. When PCBM is blended with CsPbI_3_ QD, especially after MeOAc treatment, a new characteristic peak at 1663 cm^−1^ appears, which may be attributed to the bonding between the C=O group of the PCBM molecule and CsPbI_3_ QD^50^.

Moreover, the top-view SEM images in Supplementary Fig. 16 clearly exhibit very similar morphology in the control and hybrid films, showing that both films are dense with similar surface roughness. Atomic force microscopy (AFM) height images in Supplementary Fig. 17 indicate that the hybrid film is smoother than the control. Both films have similar surface topography with the roughness of 7.3 nm for the control film and 7.1 nm for the hybrid film, suggesting that the PCBM addition did not significantly alter the QD film morphology.

### Flexible QD photovoltaics

**Fig. 5:**
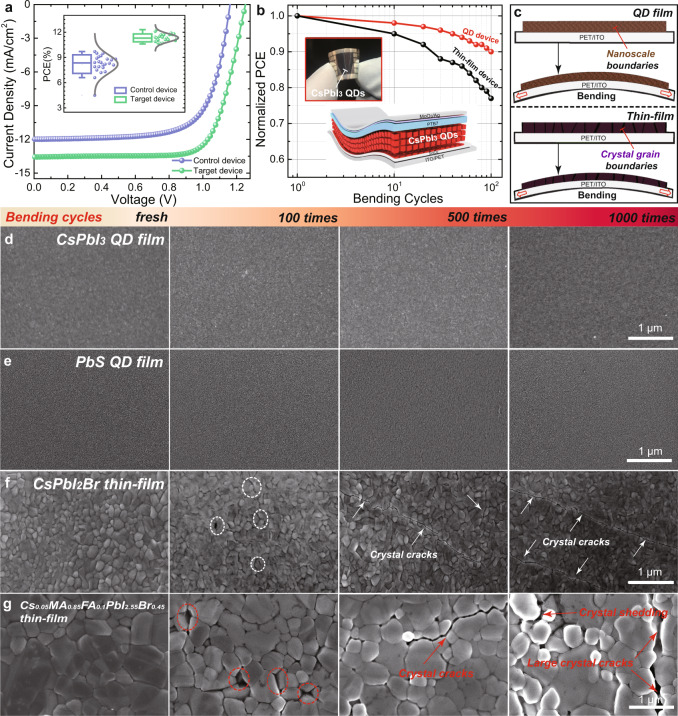
Flexible solar cells and mechanical stability. **a** *J*–*V* curves of the flexible champion device measured under AM 1.5 G solar irradiation at 100 mW/cm^2^ (inset: schematic diagram of flexible device architecture and PCE distribution of the flexible devices). **b** PCE of CsPbI_3_ QD solar cell and CsPbI_2_Br thin-film solar cell as a function of bending cycles (inset: photograph of the flexible QD solar cell). **c** Schematic diagram of the bending mechanical testing of QD and thin-film on a flexible substrate. **d**–**g** Top-view SEM images of the CsPbI_3_ QD film, PbS QD film, CsPbI_2_Br, and mixed perovskite thin-film fabricated on PET/ITO (2.5 × 2.5 cm) with mechanical bending (curvature radius of 0.75 cm), all films have a similar thickness between 300 and 400 nm.

The main goal of the HIA strategy is to provide a better ETL/QD interface to exploit the flexible nature of QD devices. To explore the advantages of the HIA strategy in fabricating efficient QD flexible solar cells, we fabricated perovskite QD flexible solar cells with the same structure for a rigid device using conventional PET (polyethylene terephthalate)/ITO substrates. Figure 5a and Table 1 presents the optimized *J*–*V* characteristics and PCE distribution of the control and target flexible devices, both the efficiency and device reproducibility are significantly improved using HIA modification, with a record PCE of 12.3%, greatly outperforming both control and previous reports (Supplementary Table 2). What is more exciting is that this HIA strategy enables us to practically demonstrate the better suitability of the perovskite QDs on flexible photovoltaics compared to extensively studied thin-film materials^51–53^. Figure 5b shows the performance comparison between all-inorganic CsPbI_2_Br perovskite bulk film and QD device. It should be noted that the preparation of all-inorganic CsPbI_3_ bulk film needs a high-temperature (>320 °C) annealing process^54,55^, which will damage the PET flexible substrate. Therefore, we select CsPbI_2_Br with a decreased annealing temperature of ~100 °C. As shown in Fig. 5b, after 100 bending cycles at the radius of 0.75 cm, the QD devices retained 90% of the initial value, while only ~75% of initial efficiency remained for the CsPbI_2_Br thin-film based device. The small reduction may be attributed to the degradation of the rigid electrode ITO or Ag contact, rather than the QD layer itself^56^. The detailed device parameters, i.e., open-circuit voltage (*V*_oc_), *J*_sc_, and fill factor (FF) for CsPbI_3_ QD and CsPbI_2_Br thin-film devices are shown in Supplementary Fig. 18.

To further investigate the degradation of device performance and understand the internal stress in films after bending, we carried out the film mechanical durability (Fig. 5c) test using SEM examination on CsPbI_3_ QD and CsPbI_2_Br thin-film as well as widely studied PbS QD film and high-efficiency Cs_0.05_MA_0.85_MA_0.1_PbI_2.55_Br_0.45_ mixed perovskite thin-film for well understanding the difference of mechanical endurance between QD and bulk thin-film. All these films were deposited on the same flexible PET/ITO substrate, as shown in Supplementary Fig. 19. We do not observe any apparent surface morphology change after bending up to 1000 times in both CsPbI_3_ (Fig. 5d) and PbS (Fig. 5e) QD films. However, crystal cracks propagate noticeably in the CsPbI_2_Br thin-film after 100 bending cycles, and gradually grow up to micrometer scale with further bending (Fig. 5f). A similar trend was found that huge cracks and even severe crystal shedding appear after 1000 bending cycles in the perovskite thin-film with larger crystalline grains (Fig. 5g). We further investigated the mechanical properties of two representative films: a CsPbI_3_ QD film and a CsPbI_2_Br thin-film, since they have a similar chemical composition. AFM peak-force model^57^ was used to measure the Young’s modulus of the films. As shown in Supplementary Fig. 20, the value of Young’s modulus is 225 MPa for the CsPbI_3_ QD film and 2.3 GPa for the CsPbBr_2_I thin-film, respectively. The value of the perovskite QD film was much lower than that of bulk thin-film, suggesting a better toughness in the perovskite QD films. These results allow us to conclude that QD films possess much better mechanical durability than bulk film since the intrinsic nanoscale boundaries and soft surface ligands in the low-dimensional QD materials are beneficial for releasing the thin-film internal stress, as described in Fig. 5c. Based on the more durable performance of QD device, this is the first report to experimentally highlight that QDs offer higher mechanical flexibility than thin-film materials, and are advantageous for flexible devices.

In summary, by developing a simple yet efficient HIA strategy, we have demonstrated high efficiency and flexible CsPbI_3_ QD solar cells for the first time. The introduction of organic molecule PCBM into the CsPbI_3_ QD layer leads to the formation of a hybrid heterojunction interfacial connecting layer, which has an important role in enhancing the QD film quality and improving the contact between the active layer and the ETL. The homogeneous modification of PCBM within the QD layer can strengthen charge transfer at both QD heterointerfaces and QD/ETL interfaces as well as suppress interfacial recombination, which are key factors governing the device efficiency. Consequently, the HIA strategy enabled us to realize CsPbI_3_ QD solar cells with an impressive efficiency of 15.1% (stabilized power output of 14.61%), demonstrating ~25% improvement compared to the control. More importantly, we also obtained flexible perovskite QD solar cells with a PCE of 12.3% assisted by the HIA strategy, as well as improved mechanical flexibility relative to similar thin-film perovskite compositions. Armed with all these results, we believe that the work demonstrated here offers a new route to improving the performance of QD photovoltaic devices and the strategy may be generalized to advance other QD-based optoelectronics.

## Methods

### Materials

Lead iodide (PbI_2_, 99.999%, Aldrich), Cesium carbonate (Cs_2_CO_3_, 99%, Aldrich), octadecene (ODE, 90%, Aldrich), oleic acid (OA, 90%, Aldrich), oleylamine (OLA, Aldrich, 70%), hexane (anhydrous, 95%, Sigma), octane (anhydrous, ≥99%, Sigma), methyl acetate (MeOAc, anhydrous, 99.5%, Sigma), chlorobenzene (CB, anhydrous, 99.8%, Sigma), PCBM (>99.9, Sigma), PTB7 (1-Materials), and tin(IV) oxide (SnO_2_, 15% in H_2_O colloidal dispersion, Alfa Aesar) were used as received without further purification unless mentioned. Glass/ITO and PET/ITO were purchased from the Zhuhai Kaivo Optoelectronic Technology Co. Ltd.

### Characterizations

The PL spectra measurements were performed at room temperature using a custom laser PL spectroscopy system (Crystal Laser, Model BLC-050-405). The laser pulse width was 130 fs, and the repetition rate was 100 MHz. The excitation wavelength for both PL and TRPL measurements is 600 nm. SEM measurements were carried out by using an FEI Nova Nano SEM 450. TEM measurements were performed by a JEOL JEM-2010 and JEOL JEM-F200 operated at 200 kV. UV–Vis absorption spectra were obtained using a U-4100 spectrophotometer (Hitachi). FTIR was performed on a Thermo Fisher FTIR6700. XPS and UPS measurements were conducted by a VG ESCALAB MK2 system with monochromatized Al Kα radiation under a pressure of 5.0 × 10^−7^ Pa. Contact angle measurements were conducted by a Data physics OCA-20 system at room temperature in an ambient atmosphere. The TA measurements were carried out under ambient conditions, the fs-TA measurements were performed on a Helios pump-probe system (Ultrafast Systems LLC) combined with an amplified femtosecond laser system (Coherent). The mechanical stability of both flexible solar cells and various flexible films were bent on a cyclic bending robot tester shown in Supplementary Fig. 18.

### CsPbI_3_ QD synthesis and purification

1 g PbI_2_ and 50 mL ODE were added into 250 mL three-neck flask and under vacuum for 60 min at 110 °C. The flask was then filled with N_2_ and heated under protection by N_2_ flow, following by the addition of 5 mL of OA and 5 mL of OLA. When the temperature further reaches 165 °C, 8 mL Cs-oleate^32^ (0.0625 M) precursor was swiftly injected into the reaction mixture. The mixture became dark red rapidly, and after 5 s the reaction was quenched by an ice bath. During the purification process, cubic-phase QDs were extracted by anhydrous MeOAc. Firstly, the synthesized CsPbI_3_ QDs were separated into eight parts and each one precipitated by adding 24 mL MeOAc (ratio of QD reaction solution: MeOAc is 1:3), then centrifuged at 8000 rpm for 5 min. The precipitation in each centrifuge tube was re-dispersed in 3 mL hexane, precipitated again with 2-fold volume MeOAc (6 mL), and centrifuged at 10,000 rpm for 5 min. The QDs were then re-dispersed in 20 mL hexane total and centrifuged again at 4000 rpm for 5 min to remove excess PbI_2_ and Cs-oleate. The supernatant was kept at 4 °C for 12 h in the dark to precipitate excess Cs-oleate and Pb-oleate. After cooling, the QD solution was centrifuged again at 4000 rpm for 5 min to obtain the final product. The final product was dried with N_2_ gas and re-dispersed into octane and chlorobenzene with a concentration of 70 mg/mL for each. For the preparation of the PCBM/CsPbI_3_ QD hybrid blend solutions, PCBM solids were added directly into CsPbI_3_ QD chlorobenzene solution, the PCBM: CsPbI_3_ ratio is controlled to be 5 mg/mL:70 mg/mL, and the hybrid blend solution was further stirred in N_2_ filled glovebox for at least 4 h.

### CsPbI_3_ QD solar cell fabrication and characterization

The SnO_2_ nanoparticle solution with a diluted weight concentration of 2.5% was spin-coated onto pre-patterned ITO glass substrates and annealed at 120 °C for 30 min in ambient condition. Then the SnO_2_ film was treated with O_2_ plasma for 2 min before the following layers would be deposited. For the control device fabrication, an ultra-thin PCBM layer was prepared by spin-coating PCBM CB solution (5 mg/mL) at 5000 RPM for 30 s. CsPbI_3_ QD layer (70 mg/mL in octane) or PCBM/CsPbI_3_ QD (5/70 mg/mL in chlorobenzene) layers were deposited by spin-coating at 1000 RPM for 30 s, treated with pure anhydrous MeOAc for 20 s. The total thickness was controlled by varying the number of deposited layers. PTB7 solution (10 mg/mL in chlorobenzene) was spin-coated on top QD films at 2000 rpm as HTLs. Finally, 10 nm MoO_3_ and 100 nm Ag electrode were prepared by thermal evaporation. For the target device fabrication, QD/PCBM hybrid solution was spin-coated on the SnO_2_ layer, treated with anhydrous MeOAc. The total thickness was control by varying deposited layers of hybrid and pure QD layers. The other layers were prepared by using the identical recipe with those of the control device. For the flexible device fabrication: SnO_2_ NP solution was spin-coated on O_2_-plasma-treated ITO/PET substrates and annealed at 120 °C for 30 min in ambient. Other layers were fabricated by using the identical recipe with that of the target device.

Both rigid and flexible QD solar cell devices were tested on a Newport AAA solar simulator (94023A-U) with xenon lamp in a glove box at room temperature using Keithley 2400 (*I*–*V*) digital source meter. The intensity of the solar simulator was calibrated to 100 mW/cm^2^ AM 1.5 G by a standard silicon cell with a KG-5 filter. *J*–*V* scans were measured from forward bias to reverse bias step and from reverse bias to forward (−1.3 V → 1.3 V, step 0.0125 V, scan rate: 0.1 V/s). The devices were masked with a black metal aperture to define an active area of 0.072 cm^2^. The stabilized power output (SPO) of devices was measured by holding the illuminated devices at a constant voltage near the maximum power point of the *J*–*V* scan and recording the continuous current output in the meantime. EQE measurements were performed utilizing an IEC 60904-8 international standard certified EQE equipment (Solar Cell Scan 100, Zolix Instruments Co. Ltd.). All these tests were performed in a glove box at room temperature.

### CsPbI_2_Br, PbS QD, and mixed perovskite film fabrication

CsPbI_2_Br thin-film devices were prepared according to reported methods^58^ with slight modifications. A solution of 1.2 M CsI, 0.6 M PbI_2_, and 0.6 M PbBr_2_ was dissolved in DMSO to obtain a precursor solution, which was spin-coated on the PET/ITO substrate at 1500 rpm for 15 s and at 4500 rpm for 45 s. In the spin-coating process of the second step, after 30 s, the MeOAc antisolvent was applied rapidly. Then, the film was heat-treated at 150 °C for 1 min. PbS QD films were fabricated using i-PbS ink^15^ spin-coated on the PET/ITO substrate at 2500 rpm for 40 s without any further treatment. Perovskite thin films were prepared according to reported methods^48^. A precursor solution of 1.4 M Cs_0.05_FA_0.80_MA_0.15_PbI_2.55_Br_0.45_ was dissolved in DMSO/DMF (volume ratio 4/1) was spin-coated on the PET/ITO substrate at 2000 rpm for 10 s and at 4000 rpm for 30 s. In the spin-coating process of the second step, after 20 s, the chlorobenzene antisolvent was applied rapidly. Then, the film was heat-treated at 100 °C for 10 min.

### Reporting summary

Further information on experimental design is available in the Nature Research Reporting Summary linked to this paper.

## Supplementary information

Supplementary Information

Solar Cells Reporting Summary

## Data Availability

Data that support the findings within this work are available from the corresponding author upon reasonable request.
