# Supplementary material for: Flexible and efficient perovskite quantum dot solar cells via hybrid interfacial architecture
Source: Nat Commun. 2021 Jan 20;12:466. doi: 10.1038/s41467-020-20749-1 (PMC7817685; doi:10.1038/s41467-020-20749-1)
Supplement: Supplementary file 1 — Supplementary Information [file 41467_2020_20749_MOESM1_ESM.pdf]

# Flexible and efficient perovskite quantum dot solar cells *via* hybrid interfacial architecture

Long Hu,[1,2,3] Qian Zhao,[4,5] Shujuan Huang,[3] Jianghui Zheng,[6,7] Xinwei Guan,[1] Robert Patterson,[6] Jiyun Kim,[1] Lei Shi,[6] Chun-Ho Lin,[1] Qi Lei,[1] Dewei Chu,[1] Wan Tao,[1] Soshan Cheong,[8] Richard D. Tilley,[8] Anita W. Y. Ho-Baillie,[6,7] Joseph M. Luther,[5] Jianyu Yuan,[2,*] and Tom Wu[1]

[1]School of Materials Science and Engineering, University of New South Wales (UNSW), Sydney, NSW, 2052, Australia.

[2]Institute of Functional Nano & Soft Materials (FUNSOM), Jiangsu Key Laboratory for Carbon-Based Functional Materials & Devices, Soochow University, Suzhou, Jiangsu 215123, P. R. China.

[3]School of Engineering, Macquarie University, Sydney, NSW 2109, Australia

[4] School of Materials Science and Engineering, Nankai University, Tianjin 300350, China.

[5] National Renewable Energy Laboratory, Golden, CO 80401, USA.

[6]Australian Centre for Advanced Photovoltaics, University of New South Wales, Sydney, Australia

[7] School of Physics, University of Sydney Nano Institute, The University of Sydney, NSW, 2006, Australia.

[8]Electron Microscope Unit, Mark Wainwright Analytical Centre, UNSW, Sydney, NSW, 2052, Australia.

*To whom correspondence should be addressed. E-mail: jyyuan@suda.edu.cn

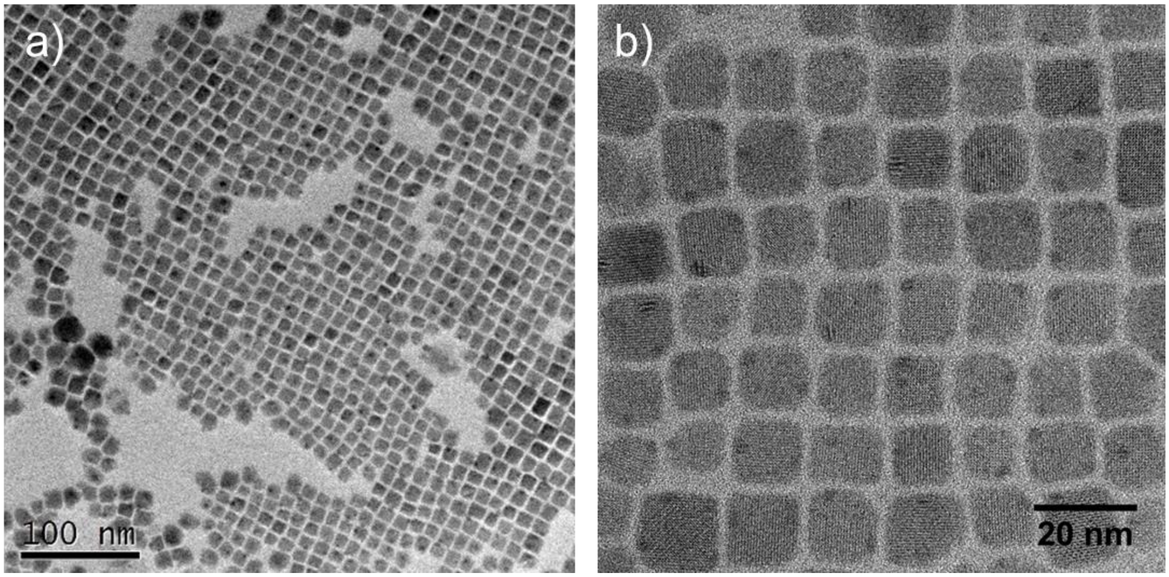


**Supplementary Figure 1. Morphology of the QDs.** The TEM images of $CsPbI_3$ QDs (a) control and (b) PCBM/QD hybrid solutions.

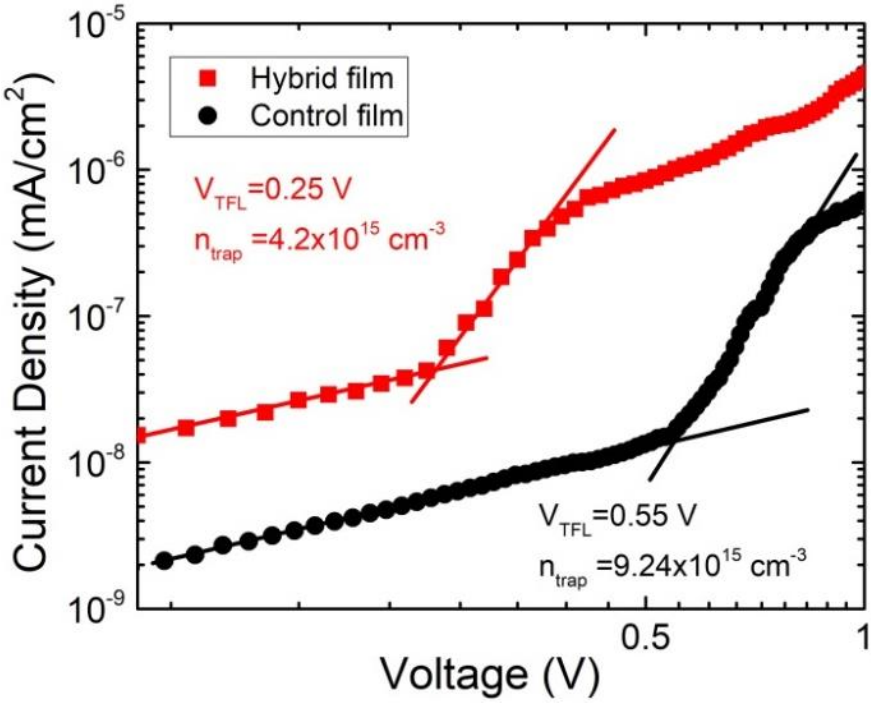


**Supplementary Figure 2. Trap state density investigation of $CsPbI_3$ QDs.** Dark current-voltage measurements of the electron-only devices for the control $CsPbI_3$ QD and PCBM/ $CsPbI_3$ QD hybrid films with a device structure of ITO/$SnO_2$/active layer/PCBM/Al.

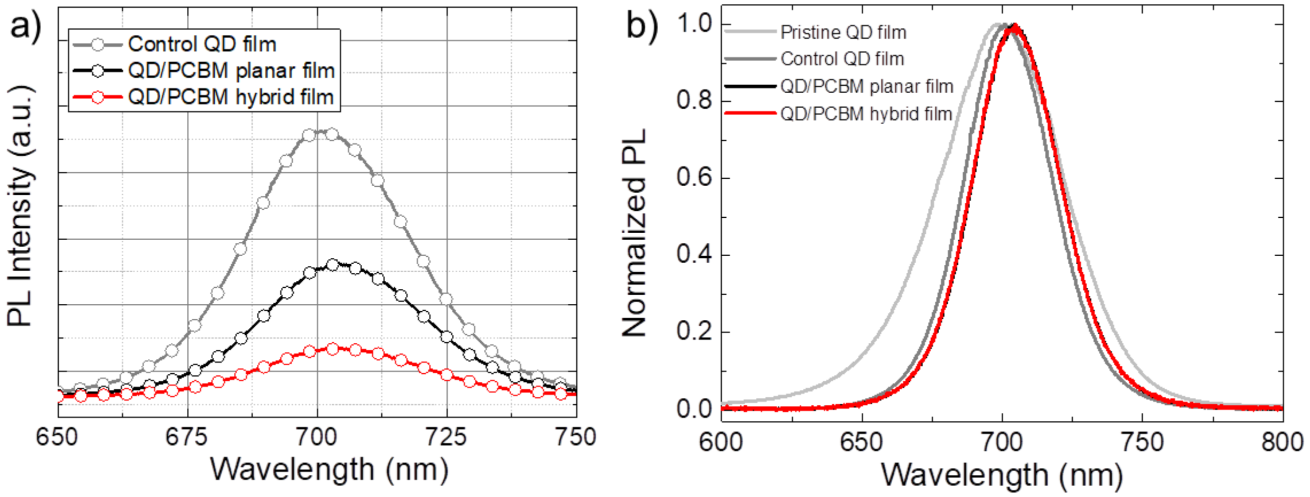


**Supplementary Figure 3. PL measurements on $CsPbI_3$ QD film under varying conditions.** (a) Steady state PL spectra of control QD, PCBM/QD planar and PCBM/QD hybrid films; (b) normalized Steady state PL spectra of pristine QD, control QD, PCBM/QD planar and PCBM/QD hybrid films, the excitation power density is 2.0 $mW/cm^2$.

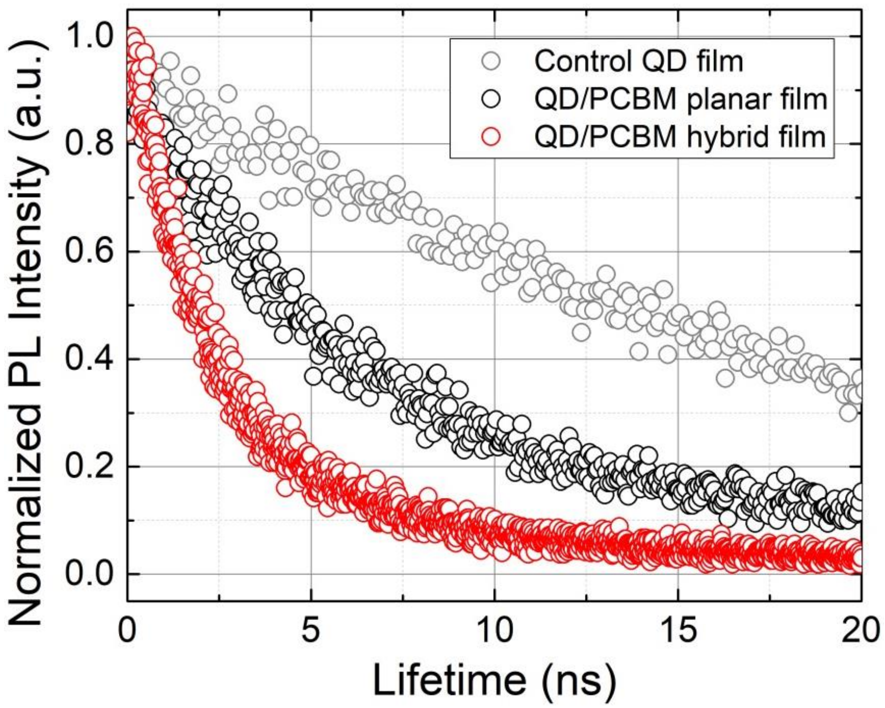


**Supplementary Figure 4**. **Time-resolved PL measurements on $CsPbI_3$ QD film.** Control QD, PCBM/QD planar and PCBM/QD hybrid films, the excitation fluence is 1.5 μJ/cm$^2$/pulse.

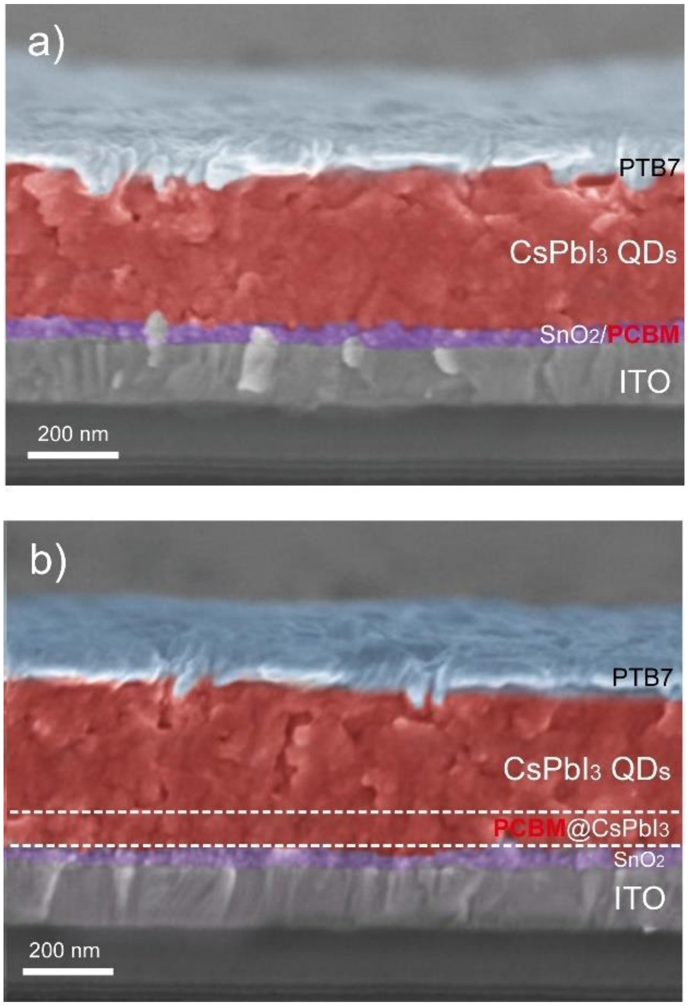


**Supplementary Figure 5. Thickness analysis in cross-sectional SEM images.** (a) control and (b) target devices, it should be noted that coloring the layer of SnO2/PCBM and PCBM@$CsPbI_3$ is purely illustrative deriving from their deposition parameters.

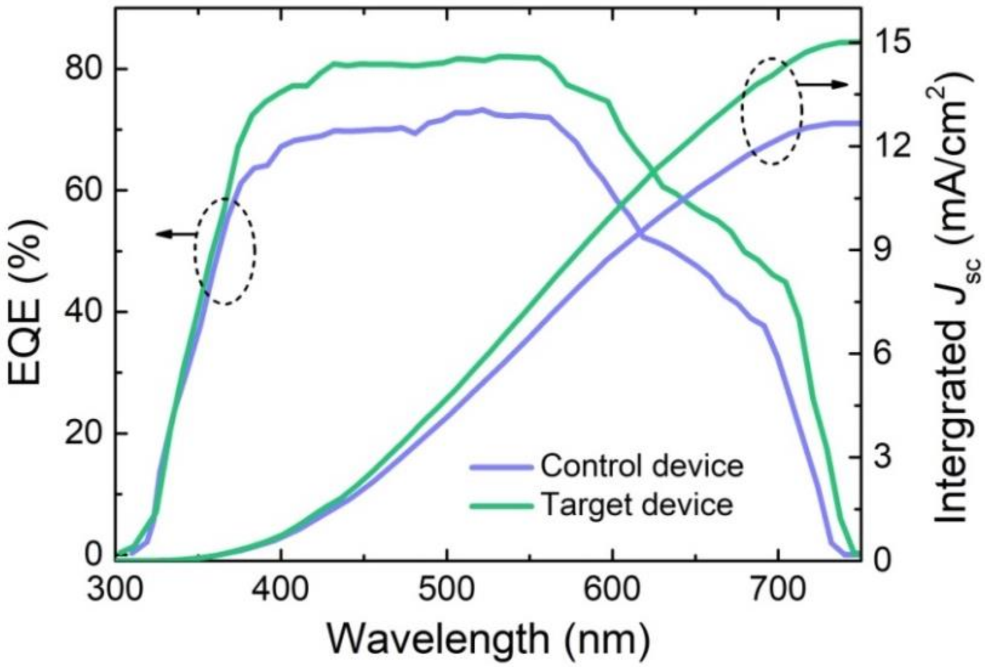


**Supplementary Figure 6**. **Photoresponse of QD solar cells.** EQE and Integrated $J_{sc}$ of the control and target $CsPbI_3$ QD solar cells.

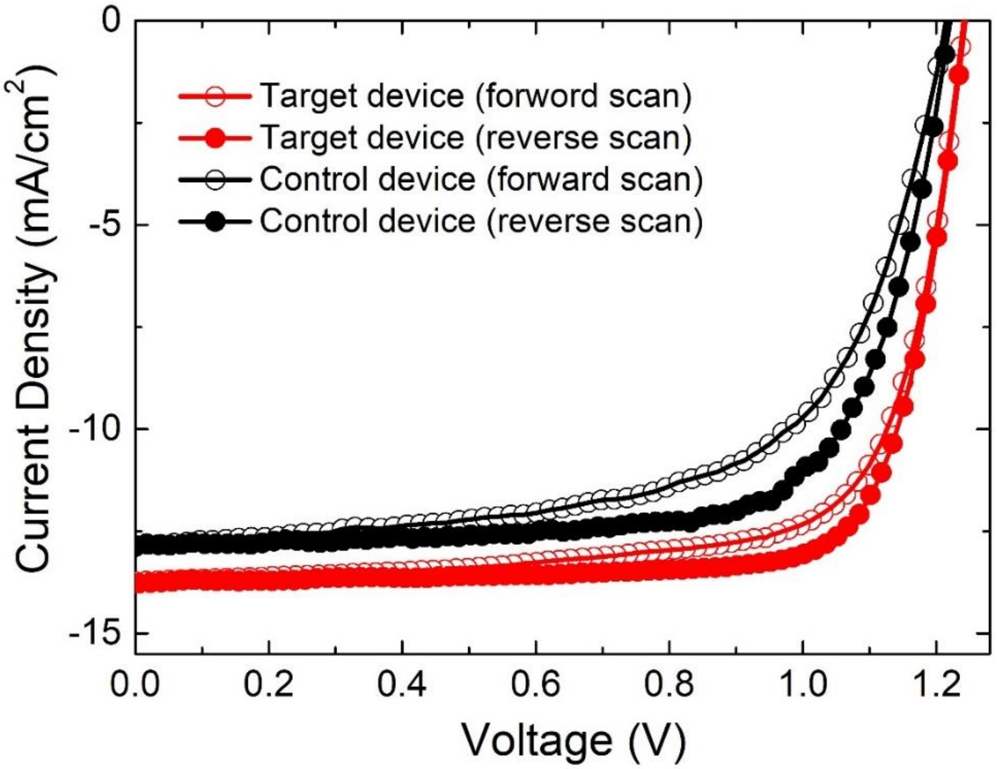


**Supplementary Figure 7**. **Hysteresis analysis of QD solar cells.** The *J*-*V* curves of target and control device under reverse and forward scanning.

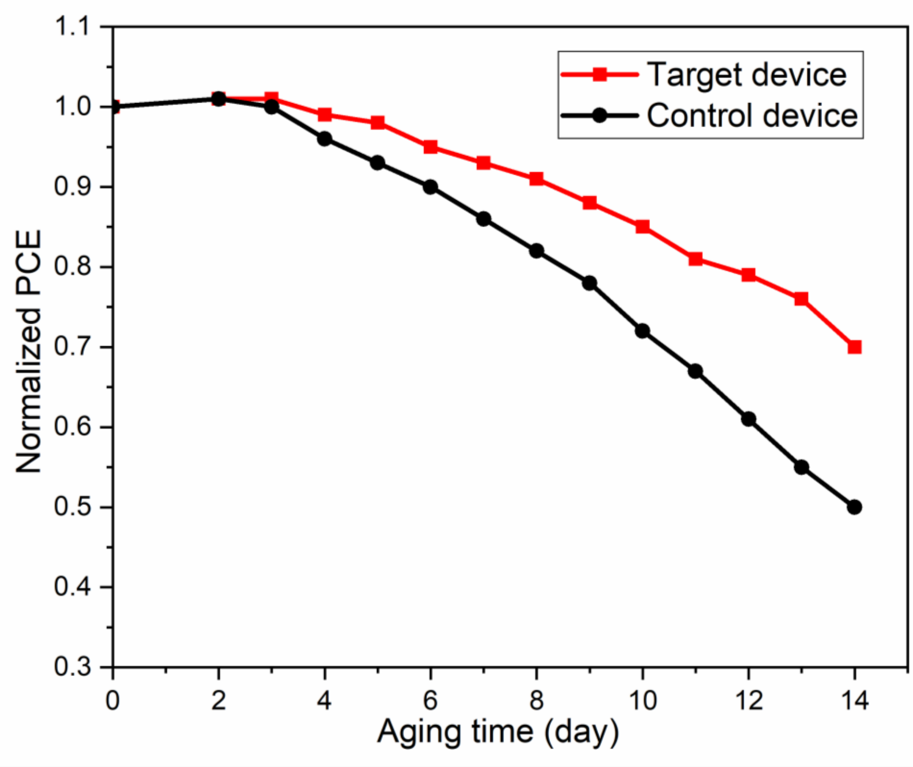


**Supplementary Figure 8. Stability of QD solar cells.** Stability of corresponding $CsPbI_3$ QD solar cells under dark conditions in a dry air atmosphere.

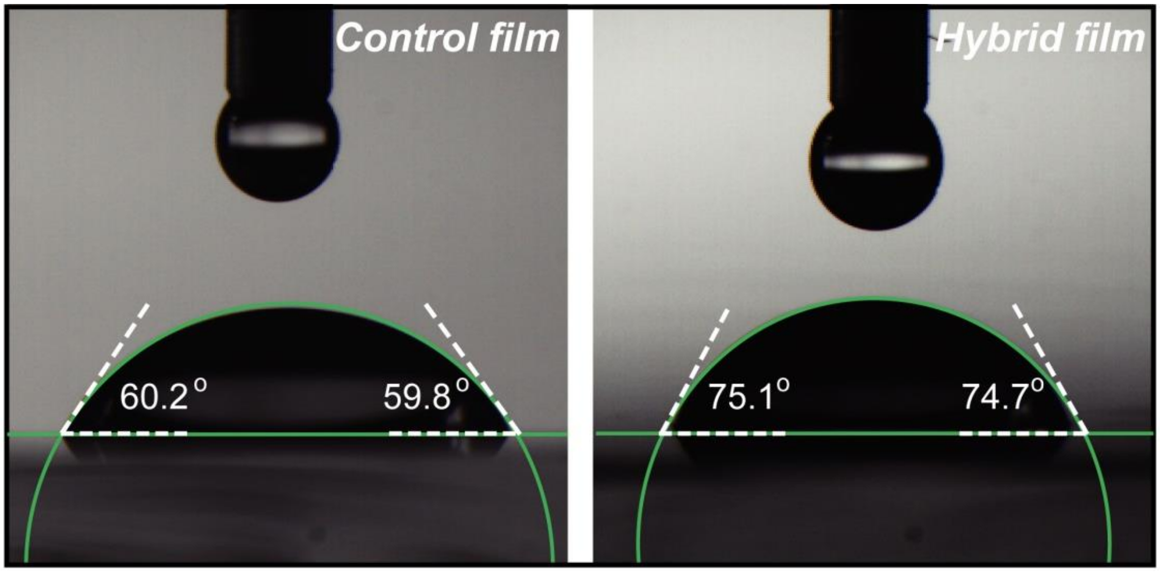

**Supplementary Figure 9. Surface properties measurements of QD films.** Contact angle of a water drop on the control and the hybrid films.

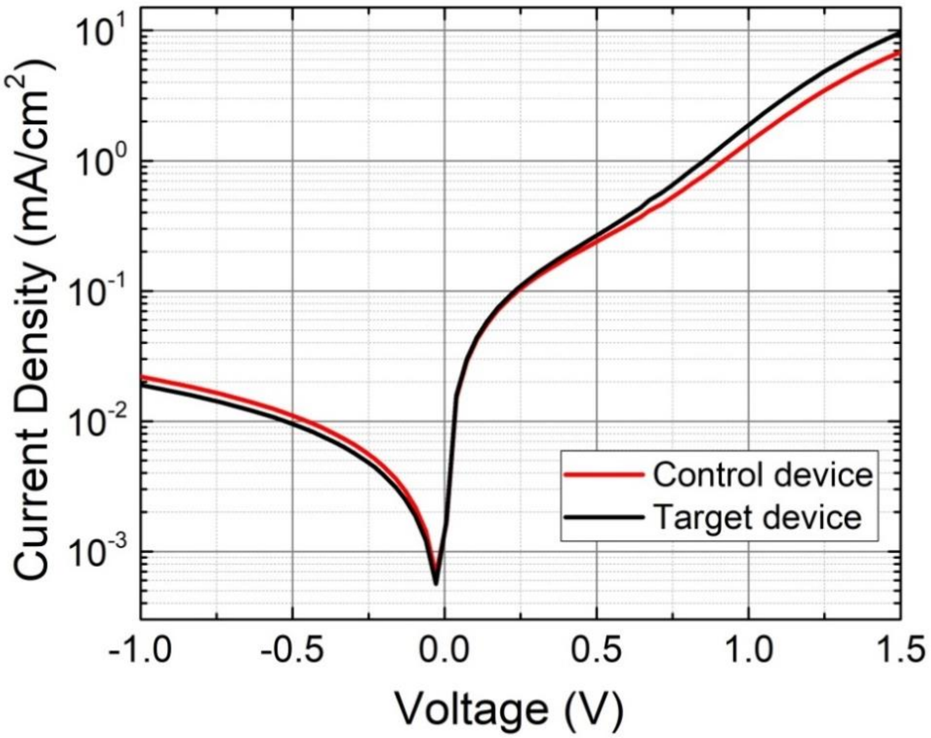


**Supplementary Figure 10. Leakage analysis of QD solar cells.** Dark current curves of the control and target devices

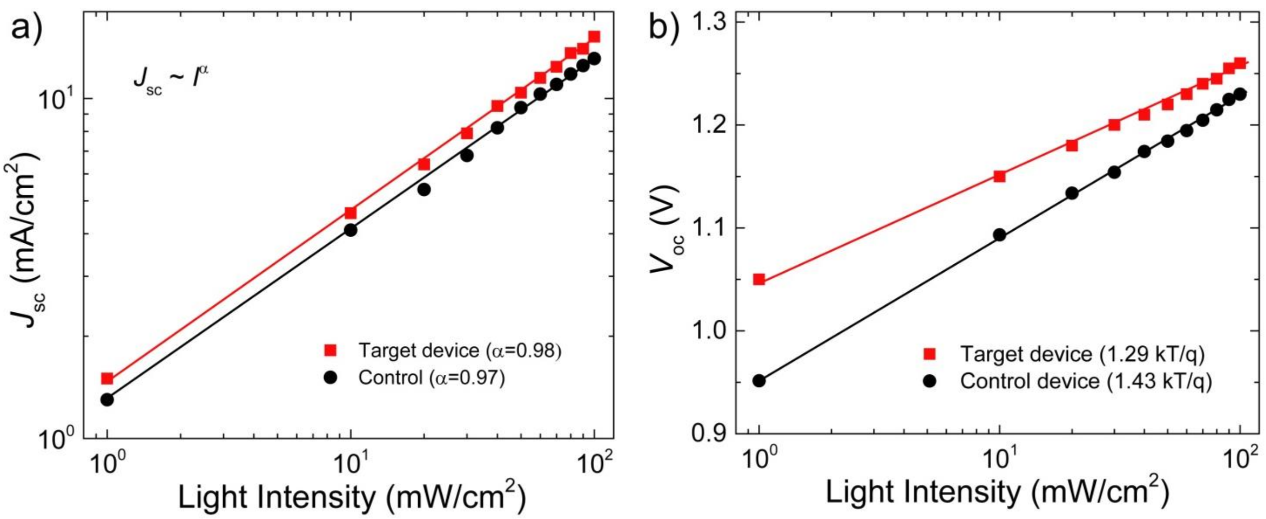


**Supplementary Figure 11. Carrier recombination investigation of QD solar cells.** (a) $J_{sc}$ and (b) $V_{oc}$ as a function of light intensity of optimized target and control $CsPbI_3$ QD solar cells.

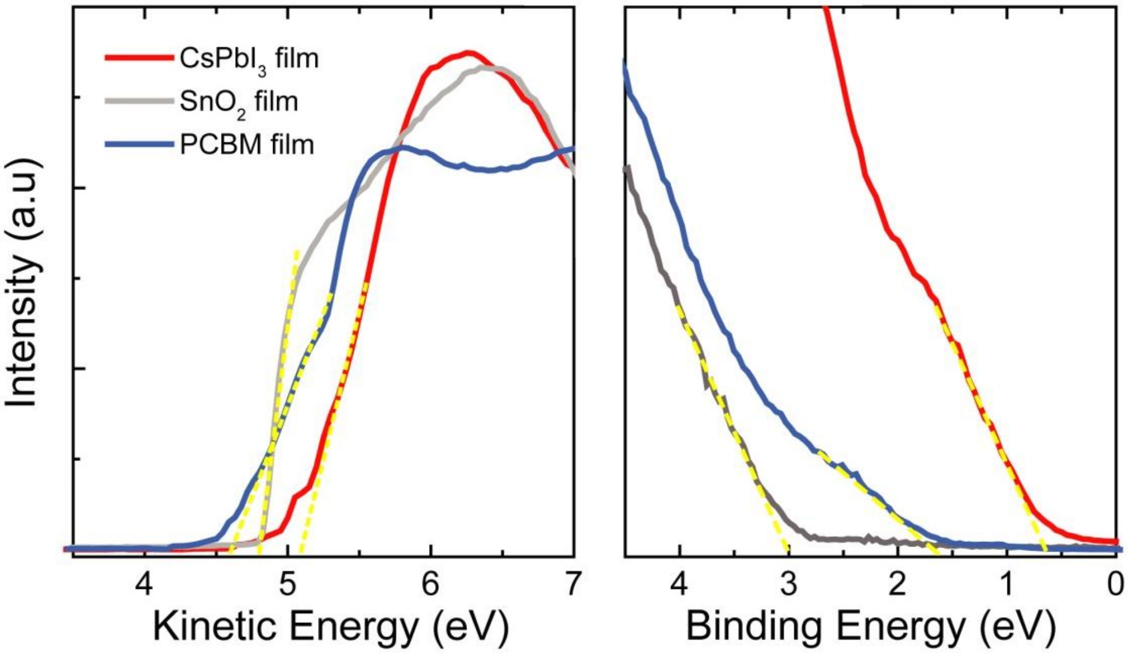


**Supplementary Figure 12. Energy level measurements.** (a) Ultraviolet photoelectron spectroscopy (UPS) cutoff edge (b) Valence band spectra of $CsPbI_3$ film, $SnO_2$ film and PCBM film from UPS measurements.

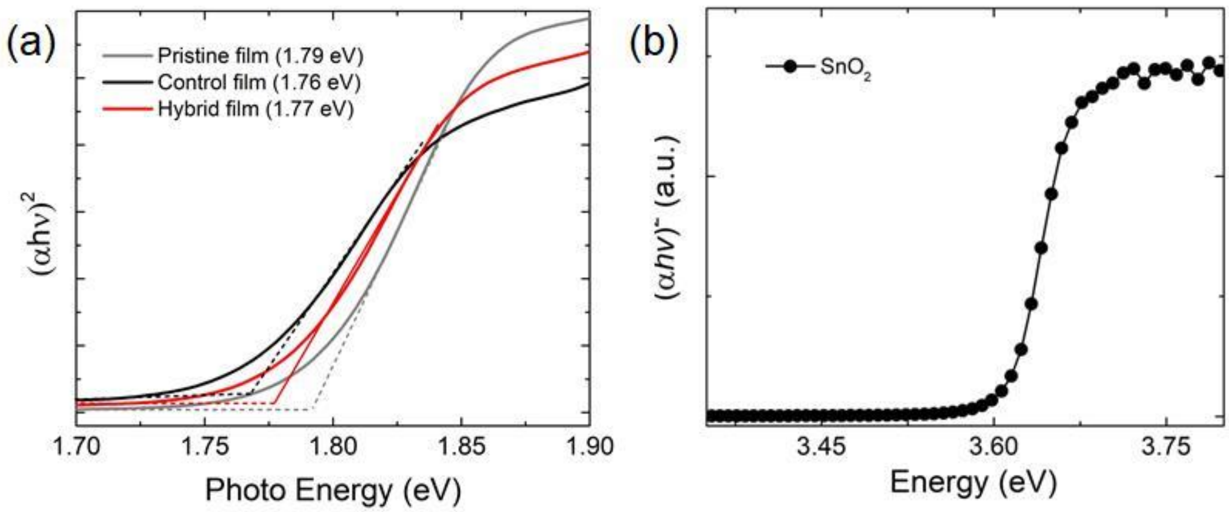


**Supplementary Figure 13. Optical absorption information.** Bandgap of (a) three types of films (b) $SnO_2$ film.

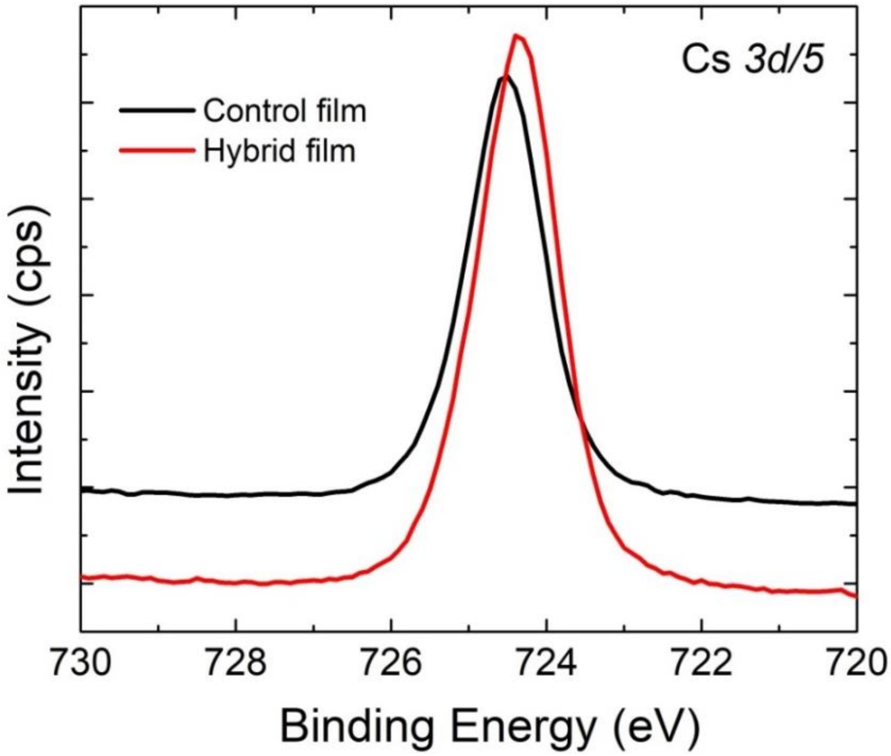


**Supplementary Figure 14. XPS measurements on QD film.** Signals of Cs 3*d*/5.

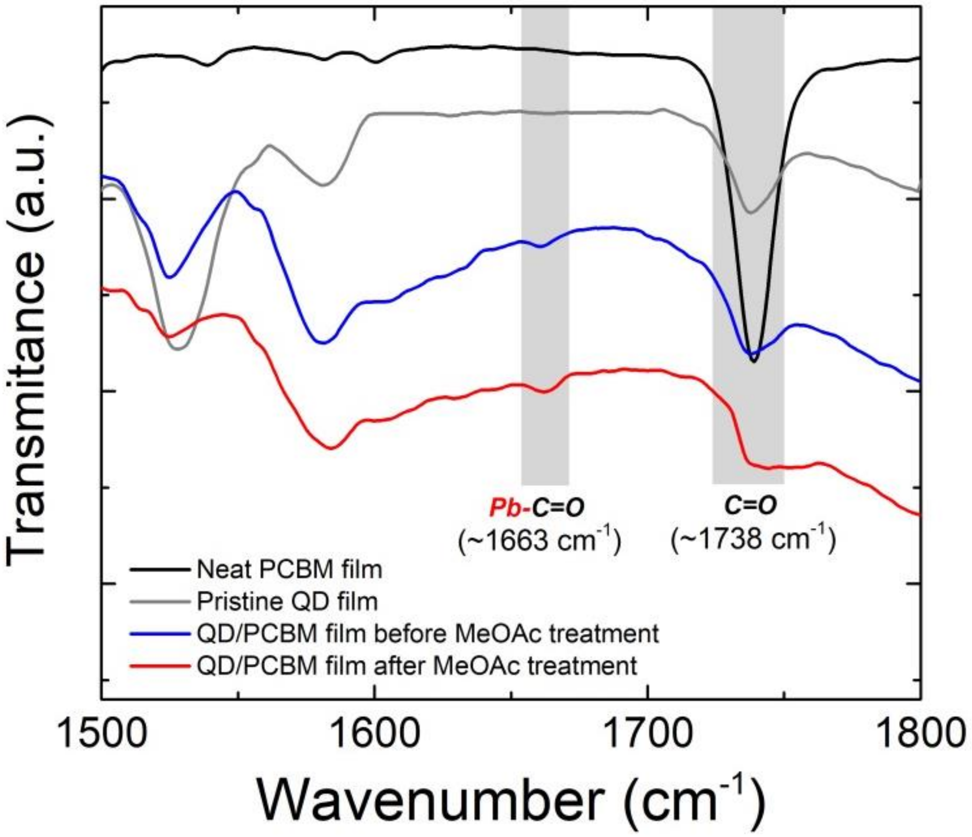


**Supplementary Figure 15. Molecular interaction analysis of PCBM-QD.** FTIR spectra of PCBM, $CsPbI_3$ QD film and PCBM-$CsPbI_3$ QD hybrid film before and after MeOAc treatment.

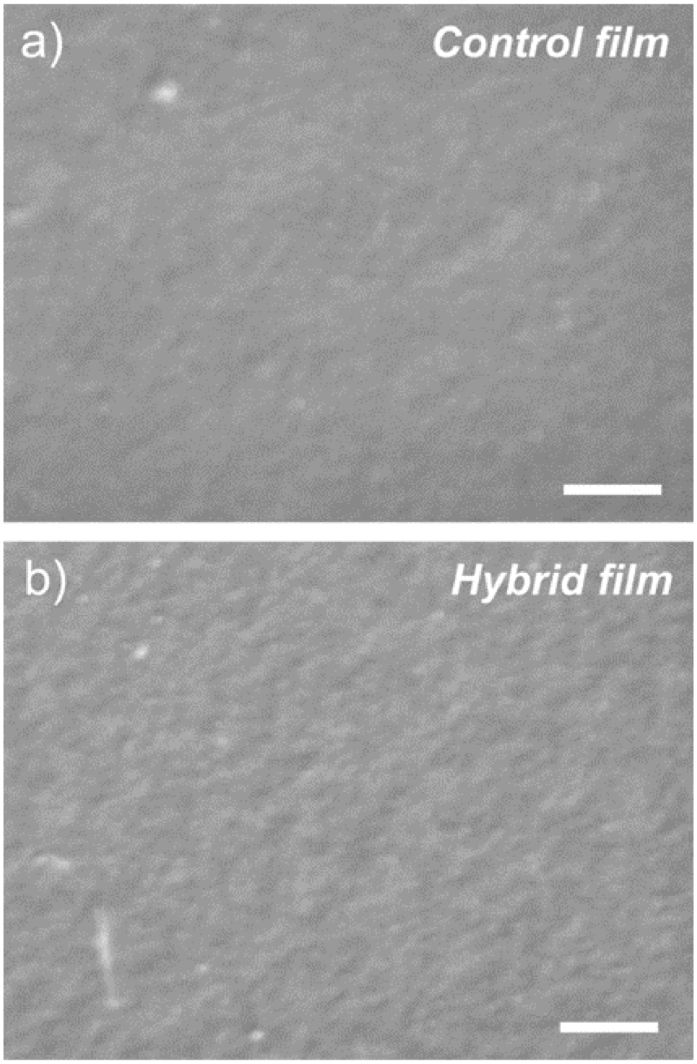


**Supplementary Figure 16. SEM Surface morphology of QD films.** Images of (a) control $CsPbI_3$ QD and (b) PCBM@$CsPbI_3$ QD hybrid films, scale bar: 1 μm

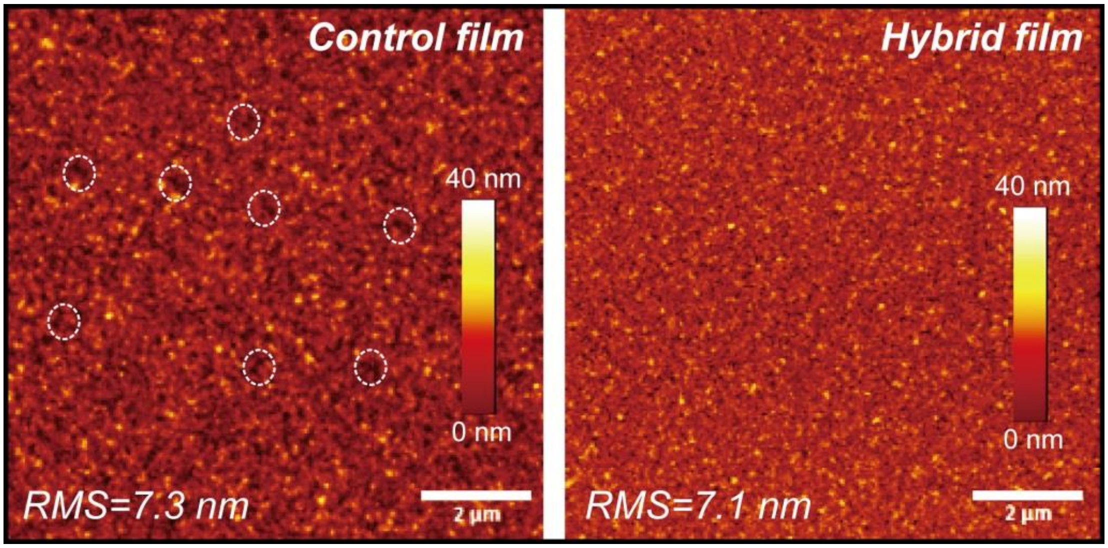


**Supplementary Figure 17. AFM Surface morphology of QD films.** Height images of the control (left) and the hybrid films (right).

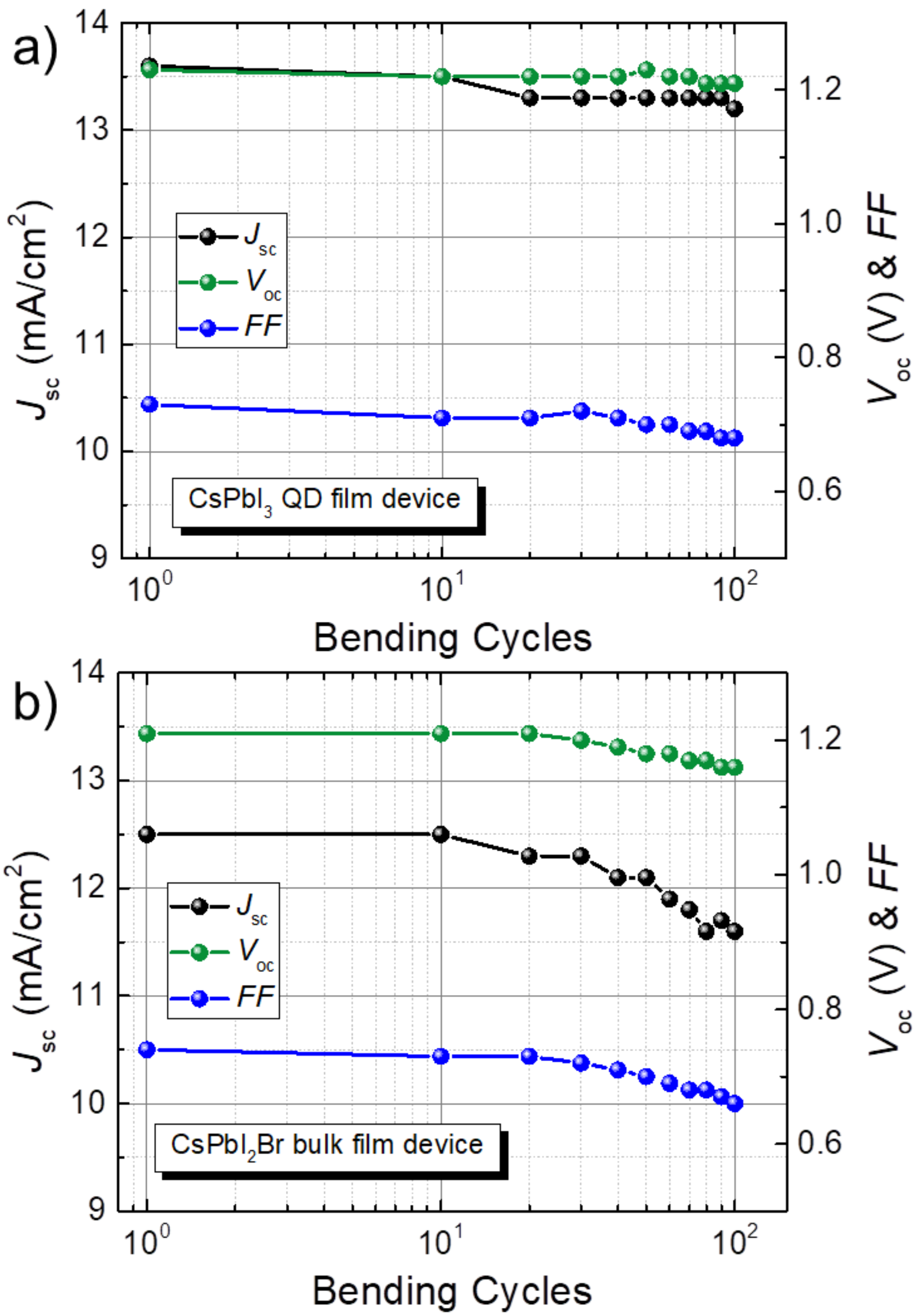


**Supplementary Figure 18. Analysis of flexible solar cells.** $J_{sc}$, $V_{oc}$ and FF of flexible $CsPbI_3$ QD (a) and $CsPbI_2Br$ bulk film (b) devices as a function of bending cycles.

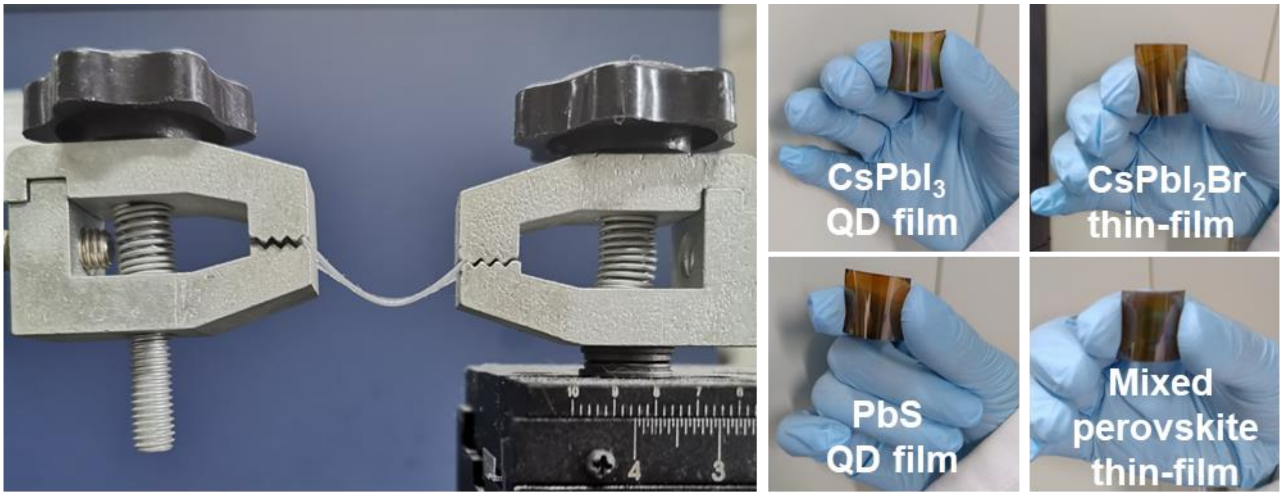


**Supplementary Figure 19. Mechanical stability measurements.** Image of bending robot and flexible films, all films were prepared on PET/ITO (2.5×2.5 cm) substrate.

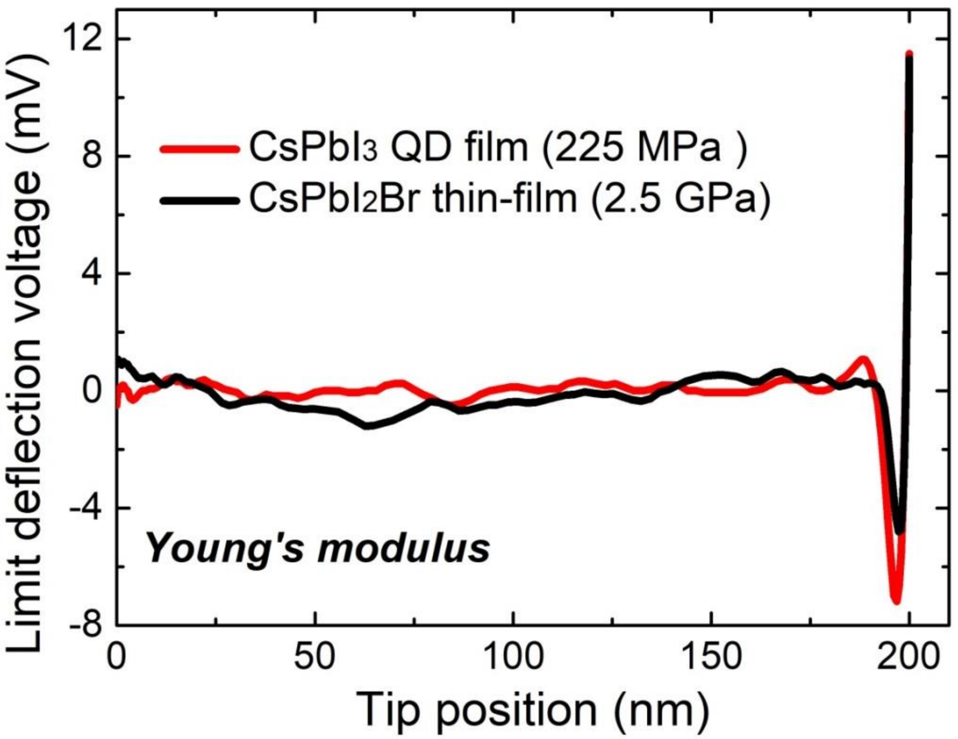


**Supplementary Figure 20. Young's modulus measurements**. Peak-force model of AFM of $CsPbI_3$ QD and $CsPbI_3Br$ films

**Supplementary Table S1.** Statistics of $CsPbI_3$ QD solar cell performance with varied QD absorber layers based on 24 devices for each condition

| **Hybrid:Control** | $V_{oc}$ **(V)** | $J_{sc}$ **(mA·cm$^{-2}$)** | **FF** | **PCE (%)** |
|---|---|---|---|---|
| 0:3 | 1.24 (1.21±0.02) | 11.4 (11.0±0.8) | 0.74 (0.72±0.03) | 10.5 (9.6±0.4) |
| 0:4 | 1.23 (1.22±0.02) | 13.6 (13.3±0.7) | 0.74 (0.73±0.02) | 12.4 (11.9±0.4) |
| 0:5 | 1.22 (1.20±0.03) | 13.4 (13.1±0.8) | 0.72 (0.72±0.03) | 11.8 (11.6±0.5) |
| 1:3 | 1.26 (1.24±0.04) | 13.9 (13.5±0.8) | 0.76 (0.74±0.03) | 13.3 (12.4±0.7) |
| 1:4 | 1.26 (1.24±0.03) | 15.2 (14.4±0.9) | 0.78 (0.76±0.03) | 15.1 (14.6±0.6) |
| 1:5 | 1.22 (1.20±0.05) | 14.4 (13.9±1.0) | 0.74 (0.72±0.04) | 13.0 (12.0±0.7) |
| 2:3 | 1.25 (1.23±0.05) | 13.9 (14.1±1.0) | 0.72 (0.73±0.04) | 13.8 (13.1±0.8) |
| 2:4 | 1.23 (1.19±0.05) | 13.8 (13.2±1.1) | 0.71 (0.69±0.04) | 12.1 (10.8±0.9) |

**Supplementary Table S2.** Summary of device information, efficiency and stability of $CsPbI_3$-QD solar cells and flexible QD solar cells reported so far.

| Device architecture | QDs | Rigid device PCE | Flexible device PCE | Reference |
|---|---|---|---|---|
| FTO/$TiO_2$/QDs/spiro-OMeTAD/$MoO_x$/Al | $CsPbI_3$ | 10.77% | - | 1 |
| FTO/$TiO_2$/QDs/spiro-OMeTAD/$MoO_x$/Al | $CsPbI_3$ | 13.4% | - | 2 |
| FTO/$TiO_2$/QDs/spiro-OMeTAD/$MoO_x$/Al | $CsPbI_3$ | 13.47% | - | 3 |
| FTO/$TiO_2$/QDs/PTB7/$MoO_3$/Ag | $CsPbI_3$ | 12.55% | - | 4 |
| FTO/$TiO_2$/QDs/PTAA/Au | $CsPbI_3$ | 11.64% | - | 5 |
| ITO/$TiO_2$/QDs/spiro-OMeTAD/$MoO_x$/Al | $CsPbI_3$ | 13.67% | - | 6 |
| FTO/$TiO_2$ /QDs /PTAA /$MoO_x$/Ag | $CsPbI_3$ | 12.30% | - | 7 |
| FTO/$TiO_2$/QDs/spiro-OMeTAD/$MoO_x$/Ag | $CsPbI_3$ | 12.40% | - | 8 |
| FTO/$TiO_2$ /QDs /PTAA/$MoO_3$/Ag | $CsPbI_3$ | 14.1% | - | 9 |
| FTO/$TiO_2$ /QDs/PTB7 /$MoO_3$/Ag | $CsPbI_3$ | 12.27% | - | 10 |
| FTO/c-$TiO_2$/QDs/spiro-OMeTAD/Au | $CsPbI_3$ | 11.87% | - | 11 |
| ITO/$SnO_2$/QDs/spiro-OMeTAD/Au | $CsPbI_3$ | 9.6% | - | 12 |
| FTO/$TiO_2$/QDs/spiro-OMeTAD/Au | $CsPbI_3$ | 14.32% | - | 13 |
| FTO/$TiO_2$ /QDs/QD:Polymer/PTAA/$MoO_3$/Ag | $CsPbI_3$ | 13.8% | - | 14 |
| FTO/$TiO_2$ /QDs /PTAA/$MoO_3$/Ag | $CsPbI_3$ | 15.2% | | 15 |
| FTO/$TiO_2$ /QDs /PTAA/$MoO_3$/Ag | $CsPbI_3$ | 14.9% | | 16 |
| PET/ITO/ZnMgO/QDs/PbS-EDT/Au | PbS | - | 9.4% | 17 |
| PEN/Ag NWs/AZO/QDs/PbS-EDT/Au | PbS | - | 9.9% | 18 |
| **Glass (PET)/ITO/ $SnO_2$/QD:PCBM/QDs/ PTB7/$MoO_3$/Ag** | **$CsPbI_3$** | **15.1%** | **12.3%** | **This Work** |

## References


1. Swarnkar A*, et al.* Quantum dot–induced phase stabilization of α-$CsPbI_3$ perovskite for high-efficiency photovoltaics. *Science* **354**, 92-95 (2016).
2. Sanehira EM*, et al.* Enhanced mobility CsPbI3 quantum dot arrays for record-efficiency, high-voltage photovoltaic cells. *Sci. Adv.* **3**, eaao4204 (2017).
3. Hazarika A*, et al.* Perovskite quantum dot photovoltaic materials beyond the reach of thin films: full-range tuning of A-site cation composition. *ACS Nano* **12**, 10327-10337 (2018).
4. Yuan J*, et al.* Metal Halide Perovskites in Quantum Dot Solar Cells: Progress and Prospects. *Joule* **4**, 1160-1185 (2020).
5. Wang Q, *et al.* m-graphene crosslinked CsPbI3 quantum dots for high efficiency solar cells with much improved stability. Adv. Energy Mater. **8**, 1800007 (2018).
6. Zhao Q*, et al.* High efficiency perovskite quantum dot solar cells with charge separating heterostructure. *Nat. Commun.* **10**, 2842 (2019).
7. Li F*, et al.* Perovskite Quantum Dot Solar Cells with 15.6% Efficiency and Improved Stability Enabled by an α-CsPbI3/FAPbI3 Bilayer Structure. *ACS Energy Lett.* **4**, 2571-2578 (2019).
8. Kim J, *et al.* Alkali acetate-assisted enhanced electronic coupling in CsPbI3 perovskite quantum dot solids for improved photovoltaics. Nano Energy **66**, 104130 (2019).
9. Ling X, *et al.* 14.1% CsPbI3 perovskite quantum dot solar cells via cesium cation passivation. Adv. Energy Mater. 9, 1900721 (2019).
10. Shi J, *et al.* Efficient and stable CsPbI3 perovskite quantum dots enabled by in situ ytterbium doping for photovoltaic applications. J. Mater. Chem. A **7**, 20936-20944 (2019).
11. Chen K, *et al.* Short-chain ligand passivated stable a-CsPbI3 quantum dot for all-inorganic perovskite solar cells. Adv. Funct. Mater. **29**, 1900991 (2019).
12. Hao M*, et al.* Ligand-assisted cation-exchange engineering for high-efficiency colloidal $Cs_{1-x}FA_xPbI_3$ quantum dot solar cells with reduced phase segregation. *Nat. Energy* **5**, 79-88 (2020).
13. Chen K*, et al.* High Efficiency Mesoscopic Solar Cells Using $CsPbI_3$ Perovskite Quantum Dots Enabled by Chemical Interface Engineering. *J. Am. Chem. Soc*. 142, 3775-3783 (2020)
14. Ji K, *et al.* High-efficiency perovskite quantum dot solar cells benefiting from a conjugated polymer quantum dot bulk heterojunction connecting layer. J. Mater. Chem. A **8**, 8104-8112 (2020).
15. Ling X*, et al.* Guanidinium-Assisted Surface Matrix Engineering for Highly Efficient Perovskite Quantum Dot Photovoltaics. *Adv. Mater.* **32**, 2001906 (2020).
16. Wang Y*, et al.* Surface Ligand Management Aided by a Secondary Amine Enables Increased Synthesis Yield of $CsPbI_3$ Perovskite Quantum Dots and High Photovoltaic Performance. *Adv. Mater.* **32**, 2000449 (2020).
17. Zhang X, Santra PK, Tian L, Johansson MB, Rensmo Hk, Johansson EM. Highly efficient flexible quantum dot solar cells with improved electron extraction using MgZnO nanocrystals. *ACS Nano* **11**, 8478-8487 (2017).
18. Zhang X, Öberg VA, Du J, Liu J, Johansson EM. Extremely lightweight and ultra-flexible infrared light-converting quantum dot solar cells with high power-per-weight output using a solution-processed bending durable silver nanowire-based electrode. *Energy Environ. Sci.* **11**, 354-364 (2018).
