# Supplementary material for: Flexible and efficient perovskite quantum dot solar cells via hybrid interfacial architecture
Source: Nat Commun. 2021 Jan 20;12:466. doi: 10.1038/s41467-020-20749-1 (PMC7817685; doi:10.1038/s41467-020-20749-1)
Supplement: Supplementary file 2 — Solar Cells Reporting Summary [file 41467_2020_20749_MOESM2_ESM.pdf]

nature research

Corresponding author(s): Jianyu Yuan

Solar Cells Reporting Summary

Nature Research wishes to improve the reproducibility of the work that we publish. This form is intended for publication with all accepted papers reporting the characterization of photovoltaic devices and provides structure for consistency and transparency in reporting. Some list items might not apply to an individual manuscript, but all fields must be completed for clarity.

For further information on Nature Research policies, including our data availability policy, see Authors & Referees.

Experimental design

Please check: are the following details reported in the manuscript?

1. Dimensions

Area of the tested solar cells
- [x] Yes — 0.072 cm2 area are used, all details are in the methods.
- [ ] No — *Explain why this information is not reported/not relevant.*

Method used to determine the device area
- [x] Yes — It has been determined by a metal mask
- [ ] No — *Explain why this information is not reported/not relevant.*

2. Current-voltage characterization

Current density-voltage (J-V) plots in both forward and backward direction
- [x] Yes — In the J-V graphs in supplementary information (Figure S7)
- [ ] No — *Explain why this information is not reported/not relevant.*

Voltage scan conditions
For instance: scan direction, speed, dwell times
- [x] Yes — J-V scans were measured from forward bias to reverse bias step and from reverse bias to forward (-1.3 V → 1.3 V, step 0.0125 V, scan rate: 0.1 V s-1). The details are given in
the methods section. Dwell time was not applied during the measurements.
- [ ] No — *Explain why this information is not reported/not relevant.*

Test environment
For instance: characterization temperature, in air or in glove box
- [x] Yes — All details about the measurement conditions are added in the methods section.
- [ ] No — *Explain why this information is not reported/not relevant.*

Protocol for preconditioning of the device before its characterization
- [x] Yes — J-V measurements were performed after the cell had undergone maximum-powerpoint tracking for 300 s. This was done to ensure that the cell reached a stable steady-state efficiency before J-V characterization.
- [ ] No — *Explain why this information is not reported/not relevant.*

Stability of the J-V characteristic
Verified with time evolution of the maximum power point or with the photocurrent at maximum power point; see ref. 7 for details.
- [x] Yes — Stabilized PCEs of both reference and target solar cells were provided in Fig. 2d
- [ ] No — *Explain why this information is not reported/not relevant.*

3. Hysteresis or any other unusual behaviour

Description of the unusual behaviour observed during the characterization
- [x] Yes — The observed hysteresis is negligible, especially in our optimized device.
- [ ] No — *Explain why this information is not reported/not relevant.*

Related experimental data
- [x] Yes — In the J-V graphs in supplementary information (Figure S7)
- [ ] No — *Explain why this information is not reported/not relevant.*

4. Efficiency

External quantum efficiency (EQE) or incident photons to current efficiency (IPCE)
- [x] Yes — In the main text, Fig 2d.
- [ ] No — *Explain why this information is not reported/not relevant.*

A comparison between the integrated response under the standard reference spectrum and the response measure under the simulator
- [x] Yes — In the Figure S6 in supplementary information
- [ ] No — *Explain why this information is not reported/not relevant.*

For tandem solar cells, the bias illumination and bias voltage used for each subcell
- [ ] Yes — *State where this information can be found in the text.*
- [x] No — We didn't make any tandem devices in this work

5. Calibration

Light source and reference cell or sensor used for the characterization	☒ Yes ☐ No	For in house measurements, a AAA solar simulator (94023A-U) with xenon lamp calibrated by using Newport certified c-Si cell.<br>*Explain why this information is not reported/not relevant.*
Confirmation that the reference cell was calibrated and certified	☐ Yes ☒ No	*State where this information can be found in the text.*<br>The reference cell is fabricated according recent report with similar photovoltaic performance
Calculation of spectral mismatch between the reference cell and the devices under test	☐ Yes ☒ No	*State where this information can be found in the text.*<br>The light spectrum used for measurements matches well with the reference silicon cell, and we did not calculate the spectral mismatch between the reference cell and the tested devices

6. Mask/aperture

Size of the mask/aperture used during testing	☐ Yes ☒ No	Stated in Method section.<br>We didn't use mask/aperture during testing since these are small area devices
Variation of the measured short-circuit current density with the mask/aperture area	☐ Yes ☒ No	*State where this information can be found in the text.*<br>We didn't measure devices with mask/aperture

7. Performance certification

Identity of the independent certification laboratory that confirmed the photovoltaic performance	☐ Yes ☒ No	*State where this information can be found in the text.*<br>The highest efficiency of 15.1% is not highest report for QD solar cells, so we didn't performed any independent certification.
A copy of any certificate(s)<br>*Provide in Supplementary Information*	☐ Yes ☒ No	*State where this information can be found in the text.*<br>We didn't performed any independent certification

8. Statistics

Number of solar cells tested	☒ Yes ☐ No	Stated in the main text.<br>*Explain why this information is not reported/not relevant.*
Statistical analysis of the device performance	☒ Yes ☐ No	Stated in the main text.<br>*Explain why this information is not reported/not relevant.*

9. Long-term stability analysis

Type of analysis, bias conditions and environmental conditions<br>*For instance: illumination type, temperature, atmosphere humidity, encapsulation method, preconditioning temperature*	☒ Yes ☐ No	Figure S8 in the supplementary information, we test the ambient stability without encapsulation in a dry air condition<br>*Explain why this information is not reported/not relevant.*
